# l-Leucine Supplementation Worsens the Adiposity of Already Obese Rats by Promoting a Hypothalamic Pattern of Gene Expression that Favors Fat Accumulation

**DOI:** 10.3390/nu6041364

**Published:** 2014-04-02

**Authors:** Thais T. Zampieri, Francisco L. Torres-Leal, Amanda B. Campaña, Fabio B. Lima, Jose Donato

**Affiliations:** 1 Department of Physiology and Biophysics, Institute of Biomedical Sciences, University of São Paulo, São Paulo 05508-000, Brazil; E-Mails: thaizampieri@gmail.com (T.T.Z.); torresleal@ufpi.edu.br (F.L.T.-L.); amanda.baron@gmail.com (A.B.C.); fabio@icb.usp.br (F.B.L.); 2 Department of Biophysics and Physiology, Federal University of Piauí, Teresina 64049-550, Brazil

**Keywords:** BCAA, amino acids, obesity, high-fat diet, energy balance, food intake, energy expenditure, hypothalamus

## Abstract

Several studies showed that l-leucine supplementation reduces adiposity when provided before the onset of obesity. We studied rats that were exposed to a high-fat diet (HFD) for 10 weeks before they started to receive l-leucine supplementation. Fat mass was increased in l-leucine-supplemented rats consuming the HFD. Accordingly, l-leucine produced a hypothalamic pattern of gene expression that favors fat accumulation. In conclusion, l-leucine supplementation worsened the adiposity of rats previously exposed to HFD possibly by central mechanisms.

## 1. Introduction

Several studies have demonstrated that supplementation with the branched-chain amino acid (BCAA) l-leucine is able to produce changes in energy balance [1,2,3,4,5]. For example, it has been shown that l-leucine supplementation prevents diet-induced obesity in rodents [1]. Thus, l-leucine has been considered a pharmaconutrient that could be used for the treatment of obesity [6,7,8]. However, in these studies [1,2,3,4,5] l-leucine supplementation was provided before the onset of obesity, at the same time the animals were exposed to a high-fat diet (HFD). Just a few studies [4,9,10] assessed the effects of l-leucine supplementation in already obese animals which is a condition more likely related to a potential use of l-leucine as a nutritional supplement for the obesity treatment. Interestingly, most of these studies did not find beneficial effects of l-leucine supplementation on the energy balance and metabolic profile in previously obese animals [4,9,10].

l-leucine potentially affects multiple organs and physiological systems [11,12]. l-leucine activates the mammalian target of the rapamycin (mTOR) intracellular signaling pathway in the skeletal muscle, the liver and adipose tissue [11,12]. More recently, hypothalamic mTOR signaling was shown to regulate the energy balance. In addition, mTOR signaling can be activated by central l-leucine administration [13]. However, it is still not well understood how oral l-leucine supplementation can affect the pattern of gene expression in the brain, especially in the hypothalamus, a critical site for the energy balance regulation. Therefore, our objective was to study rats that were exposed to HFD before they started to receive oral l-leucine supplementation. We assessed the effects of l-leucine supplementation on their energy balance and hypothalamic gene expression.

## 2. Experimental Section

### 2.1. Animals

We used male Wistar rats that were provided by the animal facility of the Institute of Biomedical Sciences, University of São Paulo (São Paulo, Brazil). The animals were maintained under standard conditions of light (12 h light/dark cycle) and temperature (22 ± 2 °C). All animal procedures were approved by the Ethics Committee on the Use of Animals of the Institute of Biomedical Sciences, University of São Paulo (approval n° 013 from 15 March 2010) and were performed according to the ethical guidelines adopted by the Brazilian College of Animal Experimentation.

### 2.2. Experimental Design

Three-week-old Wistar rats were divided into two groups according to their diet: low-fat regular rodent chow diet (Chow group, 9.4% calories from fat; Quimtia, Nuvilab CR-1, Colombo, Brazil) or a high-fat diet (HFD group, 60.6% calories from fat; PragSoluções, Jaú, Brazil). After 10 weeks, each group was redistributed into two more groups: Chow-control, Chow-l-leucine, HFD-control and HFD-l-leucine (*n* = 5–7/group). The rats were kept on their original diets, but it was added 15 g/L of l-leucine (Ajinomoto, São Paulo, Brazil) in the drinking water of l-leucine groups, whereas control groups received a solution containing a mixture of nonessential amino acids (3.4 g/L of l-alanine, 2.86 g/L of l-glycine and 4.01 g/L of l-serine; Ajinomoto, São Paulo, Brazil) in the drinking water to maintain their intake of protein isonitrogenous. During all experiments, the rats had *ad libitum* access to their experimental diets and drinking bottle. After 6 weeks of supplementation, the rats were fasted for 12 h in order to empty their gastrointestinal contents and standardize their metabolic status. Subsequently, the rats were deeply anesthetized and euthanized by decapitation, and their hypothalami were dissected for gene expression analysis. We measured the mass of epididymal, mesenteric and subcutaneous fat pads to determine the adiposity of the animals.

### 2.3. Relative Gene Expression (qPCR)

Total RNA from the whole hypothalamus were extracted with TRIzol^®^ reagent (Ambion, Carlsbad, CA, USA). Assessment of RNA quantity and quality was performed with an Epoch Microplate Spectrophotometer (Biotek^®^, Winooski, VT, USA). RNA was incubated in DNase I RNase-free (Roche, Mannheim, Germany). Reverse transcription was performed with 2 μg of total RNA with SuperScript^®^ II Reverse Transcriptase (Invitrogen, Carlsbad, CA, USA) and random primers p(dN)6 (Roche, Mannheim, Germany). Real-time polymerase chain reaction (qPCR) was performed using the StepOnePlus™ Real-Time PCR System (Applied Biosystems, Carlsbad, CA, USA) using Power SYBR Green PCR Master Mix or TaqMan^®^ Gene Expression Master Mix (Applied Biosystems, Warrington, UK). Specific primers were designed for each target gene according to sequences taken from GenBank or the literature (Table 1). The internal control gene-stability measures were calculated using the GeNorm tool. It was determined that the most stable housekeeping gene for the present study was β-2M rather than GAPDH, β-Actin, HPRT, RPL-37A or TFRC. Relative quantification of mRNA was calculated by 2^−ΔΔC^^T^. Data were normalized to β-2M expression and reported as fold changes compared to values obtained from the control group (set at 1.0).

### 2.4. Statistical Analysis

Data were compared using the two-way ANOVA (l-leucine supplementation and diet as factors) and Bonferroni posttest. A *p* value of <0.05 was considered significant in all analyses. The results are expressed as the mean ± SEM. Statistical analyses were performed using GraphPad Prism software (version 5.0; GraphPad, La Jolla, CA, USA, 2007).

## 3. Results

**Figure 1:**
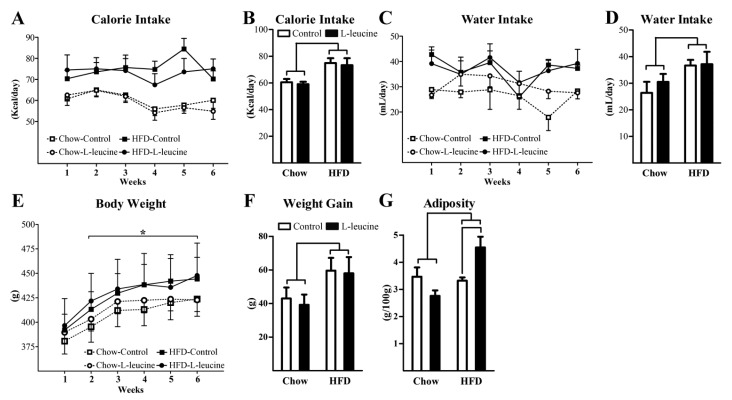
Effects of l-leucine supplementation in the energy balance regulation of rats receiving either a low- (Chow) or a high-fat diet (HFD). The weekly calorie intake (**A**); average calorie intake (**B**); weekly water intake (**C**); average water intake (**D**); body weight (**E**); weight gain rate (**F**); and adiposity (**G**) of the experimental groups. * Significantly different (*p* < 0.05) from chow groups.

During the supplementation period, the HFD groups had higher calorie intake compared to chow groups (Figure 1A,B). Chronic l-leucine supplementation did not change the calorie intake of rats receiving either the chow diet or the HFD (Figure 1A,B). Furthermore, l-leucine supplementation did not change the water intake, whereas the HFD increased the water intake independently of the supplementation (Figure 1C,D). For the rats consuming the chow diet, l-leucine supplementation in the drinking water increased total l-leucine intake 2.4-fold (0.76 ± 0.03 g/day) compared to the control group (0.31 ± 0.01 g/day). In the rats consuming a HFD, l-leucine intake increased approximately 3.2-fold in the supplemented group (0.83 ± 0.02 g/day) compared with the control HFD group (0.26 ± 0.01 g/day). The HFD groups exhibited a greater weight gain compared to groups consuming the chow diet (Figure 1E,F). l-leucine supplementation did not affect the weight gain rate of the rats (Figure 1E,F). However, the amount of body fat in l-leucine-supplemented rats consuming the HFD was increased compared to HFD-control animals (Figure 1G). In addition, we observed a significant interaction (*p* = 0.004) between l-leucine supplementation and diet factors on the adiposity data which means that the effects of l-leucine on body fat mass depends on diet.

**Table 1:** Primer sequences.

Gene of Interest	Accession No.	Forward Primer (5′-3′)	Reverse Primer (5′-3′)	Product Size (bp)
β-2M	Rn00560865_m1	*	*	*
β-Actin	NM_031144.3	AGCCTGGATGGCTACGTACA	CCTCTGAACCCTAAGGCCAA	90
GAPDH	NM_017008.4	CCGTTCAGGTCTGGGATGAC	GGGCAGCCCAGAACATCAT	76
HPRT	NM_012583.2	TTTGCTGACCTGCTGGATTAC	ACTTTTATGTCCCCCGTTGA	125
TFRC	RN01474695_m1	*	*	*
RPL-37A	RN02114291_s1	*	*	*
BCAT1	NM_017253.2	CGGCCGGACCTCAACATGAAAAGA	CCTTGTCAAACTCCGGCAGAGTGG	70
BCAT2	NM_022400.1	TCCCTGGCGTCTATGTGTGCCC	TGGAGGTCTGCCGCCTTGAAGT	63
BCKDK	NM_019244.2	AGAGCTGGCCAGGGAACGCT	TGAGGCGGACTGAGGGCTTCT	92
ORX	NM_013179.2	GCGGCCTCAGACTCCT	AGGGAGAGGCAATCCGGAGAG	70
MCH	M29712.1	ATGCTGGCCTTTTCTTTGTTT	CTTCTACGTTCCTGATGGACTT	70
NPY	NM_012614.2	CCGCCCGCCATGATGCTAGGTA	CCCTCAGCCAGAATGCCCAA	88
AgRP	NM_033650.1	GCAGAGGTGCTAGATCCACAGAA	AGGACTCGTGCAGCCTTACAC	70
POMC	NM_139326.2	ATAGACGTGTGGAGCTGGTGC	GCAAGCCAGCAGGTTGCT	75
CART	NM_017110.1	CCGAGCCCTGGACATCTACT	CCGCCTTGGCAGCTCCTT	64
ObRb	NM_012596.1	ACTTAATTTCCAAAAGCCTGAAACA	CCAGAAGAAGAGGACCAAATATCAC	83

* TaqMan^®^ Gene Expression Assay (Applied Biosystems, Carlsbad, CA, USA).

**Figure 2:**
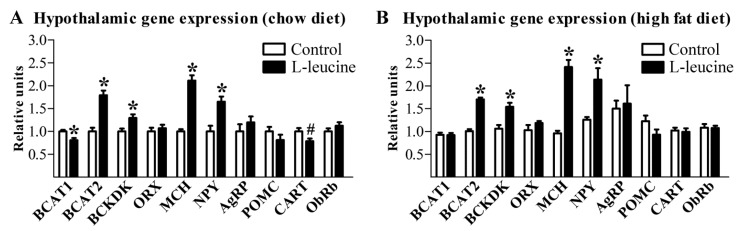
Hypothalamic mRNA expression of genes involved in the branched-chain amino acid (BCAA) metabolism and energy balance regulation. Gene expression analyses were performed in the hypothalamus of rats consuming regular chow diet (**A**) or high fat diet (**B**). * Significantly different (*p* < 0.05) from the control group. ^#^ *p* = 0.053.

Next, we assessed whether l-leucine supplementation affected the expression of genes that encode enzymes that metabolize the BCAA. The BCAT1 gene, which encodes the cytosolic form of the enzyme BCAA transaminase and is highly expressed in the brain [14], was expressed at lower levels in the l-leucine-supplemented group consuming the regular chow diet (Figure 2A). On the other hand, the genes encoding the mitochondrial form of the enzyme BCAA transaminase (BCAT2) and the branched-chain α-ketoacid dehydrogenase complex (BCKDK) showed higher expression in l-leucine-supplemented groups, regardless of diet (Figure 2A,B). Chronic l-leucine supplementation increased the hypothalamic expression of orexigenic neurotransmitters, such as melanin-concentrating hormone (MCH) and neuropeptide Y (NPY, Figure 2A,B). The cocaine and amphetamine regulated transcript (CART) showed a tendency (*p* = 0.053) towards a reduction in the l-leucine-supplemented group consuming the regular chow diet (Figure 2A). No changes in the mRNA expression of orexin (ORX), agouti-related protein (AgRP), proopiomelanocortin (POMC) or leptin receptor (ObRb) were observed with l-leucine treatment (Figure 2A,B).

## 4. Discussion

Previous studies have shown that l-leucine supplementation partially prevents the diet-induced obesity [1,2,3,4,5]. However, in these studies l-leucine supplementation and HFD were introduced in lean animals at the same time. Herein, l-leucine supplementation increased fat mass of rats previously exposed to HFD. In addition, l-leucine supplementation changed the hypothalamic expression of genes that encode enzymes that metabolize the BCAA. Furthermore, l-leucine produced an orexigenic pattern of gene expression in the hypothalamus. Therefore, a direct action of l-leucine on the hypothalamus may favor energy deposition in animals previously exposed to HFD, in opposition to the effects observed when lean animals are supplemented with l-leucine.

In the present study, l-leucine supplementation was provided in the drinking water. As a result, the supplementation was linked with water ingestion instead of food intake. In addition, the relatively low solubility of l-leucine in water precludes higher supplementation doses. Nonetheless, the chosen dose was able to produce changes in the body composition and hypothalamic gene expression of rats. Moreover, previous studies have used the same supplementation dose employed here and found significant effects of l-leucine on metabolism [1,3,5,15]. On the other hand, l-leucine supplementation in the drinking water has less chance to interfere with food palatability. Some studies found evidence of taste aversion in rodents exposed to l-leucine-rich diets [16,17]. If food palatability is negatively affected by supplementation, it may result in unintended changes in energy balance regulation.

Just a few studies investigated the effects of l-leucine supplementation in already obese animals [4,9,10,18]. For example, it was shown that l-leucine supplementation prevented the diet-induced obesity when provided before the onset of obesity. However, when l-leucine was provided in already obese mice it did not induce beneficial effects [4]. In another study, l-leucine supplementation actually increased epididymal adipocyte volume of rats previously exposed to HFD [9]. In accordance, 24-week l-leucine supplementation in old rats further increased perirenal adipose tissue mass by 45% [19]. However, in another study, in which Sprague-Dawley rats received a high-fat/high-sucrose diet for 6 weeks, l-leucine supplementation caused a significant reduction in fat mass and improved whole-body insulin sensitivity [18]. Therefore, although the effects of l-leucine on fat mass are still controversial, the metabolic condition of the organism at the beginning of supplementation may produce distinct effects on energy balance. Regarding that, most studies indicate that l-leucine may favor fat accumulation in animals that are already obese and insulin resistant. The reasons for this effect are still not well understood, but it can be possibly related to a direct effect of l-leucine on adipose tissue. l-leucine activates mTOR signaling pathway in adipocytes [20] and mTOR signaling promotes fat storage in these cells [21,22], although some authors showed that l-leucine can decrease fatty acid synthase expression and triglyceride content in cultured 3T3L1 adipocytes [23]. In addition, the adipogenic effects of mTOR activation may be intensified in previously obese and insulin resistant animals [9,21,22]. When exposed to a high-fat diet, an increased fat storage capacity in the adipose tissue may prevent fat accumulation in other tissues, such as the liver or skeletal muscle, resulting in beneficial effects on the metabolic profile and glucose tolerance in l-leucine-treated animals [1,3,4,5]. Other explanation for the beneficial effects of l-leucine on metabolism is related with the activation of SIRT1. Chronic l-leucine supplementation was shown to increase SIRT1 expression in the liver, brown adipose tissue and skeletal muscle and prevent metabolic disorders in diet-induced obese mice [5]. Human adipocytes treated with the serum from subjects consuming high dairy diets, which are rich in l-leucine, also exhibited increased SIRT1 activity and expression [24]. Other studies found a synergistic effect of l-leucine and resveratrol, a polyphenol that activates SIRT1, on the metabolism and insulin sensitivity of muscle cells and adipocytes [25,26]. Overall, these studies indicate that l-leucine may have beneficial metabolic effects independently of changes in body weight as previously demonstrated [18].

Alternatively, l-leucine can modulate the energy balance by influencing hypothalamic neurons [13]. To determine whether the brain is responsive to oral l-leucine supplementation, we assessed the expression of genes that metabolize BCAA which are sensitive to variations in l-leucine availability, at least in peripheral tissues [27,28]. In fact, the brain seems to be sensitive to an increased l-leucine intake. Hypothalamic BCAT2 expression showed a response similar to BCKDK, in which l-leucine supplementation produced a significant increase in rats consuming either low- or high-fat diets. However, the physiological significance of the opposite influence of l-leucine supplementation on the hypothalamic expression of BCAT1 and BCAT2 is unknown. Regarding genes involved with energy balance regulation, l-leucine-treated rats had an increased expression of the orexigenic neurotransmitters MCH and NPY [29]. Thus, l-leucine seems to favor a condition of positive energy balance. Although these effects did not result in changes in food intake, the hypothalamic circuitries affected by l-leucine supplementation also control the autonomic nervous system and the energy expenditure [29]. Therefore, it is likely that the higher fat mass observed in l-leucine-supplemented rats consuming the HFD may be the consequence of changes in energy expenditure rather than food intake. Interestingly, l-leucine supplementation produced similar effects on hypothalamic gene expression comparing the chow and HFD groups, although the increased adiposity was observed only when associated with HFD. The reasons for this difference are unknown, but they may be related with adaptive thermogenesis response. Previous studies have shown that l-leucine supplementation increases whole-body energy expenditure [1,4]. However, Binder *et al.* [4] observed that l-leucine supplementation increased energy expenditure only when provided before the onset of obesity. In already obese animals, l-leucine did not affect the energy expenditure [4]. Thus, it is plausible to hypothesize that the increased expression of orexigenic neurotransmitters caused by l-leucine supplementation may be counterbalanced by its stimulatory effects on energy expenditure in lean animals. In already obese rats, the central effects of l-leucine supplementation may also have favored fat mass deposition through the increased expression of MCH and NPY. However, the compensatory effects of l-leucine on energy expenditure were absent in already obese rats as previous shown [4], leading these animals to a higher adiposity. Future studies should be designed to test this hypothesis.

## 5. Conclusions

l-leucine supplementation worsened the adiposity of rats previously exposed to HFD possibly by affecting hypothalamic circuitries involved with energy balance regulation. From a therapeutic point of view, the extrapolation of our findings indicates that obese subjects may not benefit from the effects of l-leucine supplementation, although more studies in humans are still necessary to test this suggestion.
